# Whole Genome Sequencing and Analysis of Plant Growth Promoting Bacteria Isolated from the Rhizosphere of Plantation Crops Coconut, Cocoa and Arecanut

**DOI:** 10.1371/journal.pone.0104259

**Published:** 2014-08-27

**Authors:** Alka Gupta, Murali Gopal, George V. Thomas, Vinu Manikandan, John Gajewski, George Thomas, Somasekar Seshagiri, Stephan C. Schuster, Preeti Rajesh, Ravi Gupta

**Affiliations:** 1 Central Plantation Crops Research Institute, Kasaragod, Kerala, India; 2 SciGenom Labs Pvt. Ltd., Plot 43A, SDF 3rd Floor CSEZ, Kakkanad, Cochin, Kerala, India; 3 Center for Comparative Genomics and Bioinformatics, Pennsylvania State University, 310 Wartik Lab, University Park, Pennsylvania, United States of America; 4 SciGenom Research Foundation, Cochin, Kerala, India; 5 Department of Molecular Biology, Genentech Inc., South San Francisco, California, United States of America; 6 Singapore Centre on Environmental Life Sciences Engineering, Nanyang Technical University, Singapore, Singapore; UCLA-DOE Institute for Genomics and Proteomics, United States of America

## Abstract

Coconut, cocoa and arecanut are commercial plantation crops that play a vital role in the Indian economy while sustaining the livelihood of more than 10 million Indians. According to 2012 Food and Agricultural organization's report, India is the third largest producer of coconut and it dominates the production of arecanut worldwide. In this study, three Plant Growth Promoting Rhizobacteria (PGPR) from coconut (CPCRI-1), cocoa (CPCRI-2) and arecanut (CPCRI-3) characterized for the PGP activities have been sequenced. The draft genome sizes were 4.7 Mb (56% GC), 5.9 Mb (63.6% GC) and 5.1 Mb (54.8% GB) for CPCRI-1, CPCRI-2, CPCRI-3, respectively. These genomes encoded 4056 (CPCRI-1), 4637 (CPCRI-2) and 4286 (CPCRI-3) protein-coding genes. Phylogenetic analysis revealed that both CPCRI-1 and CPCRI-3 belonged to *Enterobacteriaceae* family, while, CPCRI-2 was a *Pseudomonadaceae* family member. Functional annotation of the genes predicted that all three bacteria encoded genes needed for mineral phosphate solubilization, siderophores, acetoin, butanediol, 1-aminocyclopropane-1-carboxylate (ACC) deaminase, chitinase, phenazine, 4-hydroxybenzoate, trehalose and quorum sensing molecules supportive of the plant growth promoting traits observed in the course of their isolation and characterization. Additionally, in all the three CPCRI PGPRs, we identified genes involved in synthesis of hydrogen sulfide (H_2_S), which recently has been proposed to aid plant growth. The PGPRs also carried genes for central carbohydrate metabolism indicating that the bacteria can efficiently utilize the root exudates and other organic materials as energy source. Genes for production of peroxidases, catalases and superoxide dismutases that confer resistance to oxidative stresses in plants were identified. Besides these, genes for heat shock tolerance, cold shock tolerance and glycine-betaine production that enable bacteria to survive abiotic stress were also identified.

## Introduction

Plant rhizosphere harbors numerous bacteria capable of stimulating and aiding plant growth and are termed plant growth promoting rhizobacteria (PGPR) [1]. They exert their beneficial effects through direct or indirect mechanisms. The direct mechanisms include biofertilization, stimulation of root growth, rhizo-remediation and plant stress control [2]. Indirect mechanisms primarily involve biological control comprised of antibiosis, induction of systemic resistance and competition for nutrition and niches [2]. Owing to their diverse plant growth promoting capabilities, PGPRs have become the new inoculants for biofertilizer technology [3]. To improve the biofertilizer technology, understanding the molecular mechanisms of plant growth promotion and biocontrol by rhizobacteria is important [4]. Identification of genes that contribute to the beneficial activity of rhizobacteria, besides adding to our understanding of the molecular mechanisms, will aid in developing better biofertilizers.

Next generation sequencing technologies (NGS) have enabled whole genome sequencing of bacteria and other organisms [5]. Systematic analysis of whole genome data has aided the understanding of the molecular genetics of many bacterial species [6]. Recently, NGS has been employed to study genomes of several PGPRs, mainly isolated from crop species such as wheat [7], *Miscanthus* [8], pepper [9]. PGPRs from soil have also been sequenced directly [10]. However, thus far, genome sequences of PGPRs isolated from plantation crops, particularly from coconut, cocoa and arecanut, have not been reported.

Coconut (*Cocos nucifera* L.), cocoa (*Theobroma cacao* L.) and arecanut (*Areca catechu* L.) are three important plantation crops grown in 2.2 million hectares in India. These plantation crops harbor plant-beneficial microorganisms in their rhizospheres [11]–[15] and some of these have been utilized for growth promotion [16]–[20] as they offer an opportunity for ecologically safe nutrient management [21]. In this study, we have performed deep sequencing analysis of three PGPRs, CPCRI-1, CPCRI-2 and CPCRI-3, isolated from coconut [16], cocoa [20] and arecanut [22] rhizosphere, respectively.

## Results

### PGPR strains

Soil samples collected from rhizospheres of coconut, cocoa and arecanut grown in different agro-ecological zones of India were used to isolate 1512 morphologically distinct heterotrophic bacteria [13], [15], [18], [22]. The details of places from which the soil samples were collected, soil types and their pH along with isolation media are given in Table S1. The isolates were screened *in vitro* for several important plant growth promoting functions (Table 1). The isolates that gave best results in the *in vitro* testing were then studied for plant growth promotion using rice and cowpea seeds in environmental growth chamber and green house conditions [18], [19]. They were also then tested on coconut [16], cocoa [20] and arecanut [22] seedlings grown in polybags.

**Table 1:** Biological and plant growth promotional properties of PGPR isolates.

S. No.	Attributes	CPCRI-1 (RNF 267) [16]	CPCRI-2 (KGSF20) [20]	CPCRI-3 (KtRA5-88) [22]
1.	pH tolerance levels	4.0 to 9.0	4.0 to 8.0	2.5 to 7.0
2.	Optimum pH for growth	5.0–6.0	7.0	4.5
3.	NaCl tolerance	upto 8%	upto 4%	upto 2%
4.	Temperature tolerance	15 to 40°C	15 to 40°C	30 to 40°C
5.	ACC deaminase activity	562 µmol α-ketobutyrate h^−1^ mg protein^−1^	199 µmol α-ketobutyrate h^−1^ mg protein^−1^	474 µmol α-ketobutyrate h^−1^ mg protein^−1^
6.	Phosphate solubilization	217 µg ml^−1^	99.8 µg ml^−1^	82 µg ml^−1^
7.	IAA production	2.4 µg ml^−1^	1.5 µg ml^−1^	1.2 µg ml^−1^
8.	Growth on N-free agar medium	–	Growth observed	–
9.	Chitinase activity	–	–	–
10.	β-1,3-glucanase activity	20 µg glucose min^−1^ mg protein^−1^	7.8 µg glucose min^−1^ mg protein^−1^	2.4 µg glucose min^−1^ mg protein^−1^
11.	Salicylic acid production	–	6.1 µg ml^−1^	–
12.	Siderophore production	–	6 mm	11 mm

– indicates no growth/production/activity.

Three PGPRs designated CPCRI-1 (RNF-267 from coconut) [16], CPCRI-2 (KGSF-20 from cocoa) [15], [20] and CPCRI-3 (KtRA5-88 from arecanut) [22] were selected for further studies. All the three isolates had rod shape morphology and were negative for Gram's staining. CPCRI-1 showed good phosphate solubilizing capacity and promoted growth of coconut seedlings [16]. CPCRI-2 was capable of promoting growth of cocoa seedlings [20]. CPCRI-3, isolated from arecanut rhizosphere, was able to tolerate low pH and possessed plant growth promoting attributes [22]. The plant growth promotion traits of the three isolates are summarized in Table 1. The morphological, biochemical and physiological attributes of the three PGPRs are summarized in Table S2. Given the beneficial attributes of the three PGPRs, we chose to characterize them further at the genomic level.

### Whole genome shotgun sequencing and assembly

We performed shotgun multiplexed sequencing of the genomes of CPCRI-1, CPCRI-2 and CPCRI-3 using the 454-sequencing platform. We obtained >300,000 quality-filtered reads each for CPCRI-1 and CPCRI-3 with an average read length of 465 bp and 421 bp, respectively. For CPCRI-2, we obtained >150,000 quality filtered reads with an average read length of 408 bp (Table 2). We assembled the sequencing reads for each of the three genomes using GS *de novo* assembler version 2.6 [23]. Of the total reads obtained ∼90% were assembled into contigs corresponding to each of the genomes. For CPCRI-1, 350,636 reads were assembled into 39 contigs (N50 of 242,562 bp; longest contig length of 730,806 bp; mean contig length of 114,755 bp) for a total of 4,475,442 nucleotides at ∼30× coverage. The 144,293 reads obtained for CPCRI-2, were assembled into 101 contigs (N50 of 89,849 bp; longest contig length of 282,342 bp; mean contig length of 52,329 bp) for a total of 5,285,206 nucleotides at ∼12× coverage. In the case of CPCRI-3, the 313,271 reads obtained were assembled into 47 contigs (N50 of 161,752 bp; longest contig length of 529,776 bp; mean contig length of 99,348 bp) for a total of 4,669,355 nucleotides at ∼30× coverage (Table 2, Fig. S1).

**Table 2:** Genome assembly statistics.

	CPCRI-1	CPCRI-2	CPCRI-3
# of reads	361,881	158,071	326,453
Average read length (bp)	465	408	421
# of bases (bp)	168,202,148	64,520,437	137,575,981
# of reads assembled	350,636 (96.9%)	144,293 (91.3%)	313,271 (96%)
Number of contigs (> = 500 bp)	39	101	47
N50 (bp)	242,562	89,849	161,752
Average contig length (bp)	114,755	52,329	99,348
Total length of contigs (bp)	4,475,442	5,285,206	4,669,355
GC Content (%)	56.0	63.6	54.8
Average contig size (bp)	114,755	52,329	99,348
Longest contig size (bp)	730,806	282,342	529,776
Q40 plus bases (%)	99.97	99.77	99.96

The estimated genome size based on the sequence data was 4.7 Mb for CPCRI-1, 5.9 Mb for CPCRI-2 and 5.1 Mb for CPCRI-3. Phylogenetic analysis derived from comparison of 31 conserved housekeeping protein-coding genes [24] indicated that while CPCRI-1 and CPCRI-3 were members of the *Enterobacteriaceae* family, CPCRI-2 was a *Pseudomonadaceae* family member. Their estimated genome sizes are consistent with the sizes observed for other family members (Table S3 & S4). The GC content of the bacterial isolates was 56.0%, 63.6% and 54.8% for CPCRI-1, CPCRI-2 and CPCRI-3, respectively (Table 2).

### Gene prediction and annotation

Glimmer-MG [25] predicted 4056, 4637 and 4286 protein-coding genes in CPCRI-1, CPCRI-2 and CPCRI-3, respectively (Table 3 & Table S5). Consistent with this the average predicted protein coding genes size in CPCRI-1, CPCRI-2 and CPCRI-3 was found to be 972 bp, 981 bp and 951 bp, respectively. In bacteria, a robust correlation exists between the genome size and the numbers of genes it encodes [26]. A comparison of 26 published complete genomes in the *Enterobacteriaceae* family revealed an average genome size of 4.8 Mb and the coded for an average of 4655 proteins (Table S3). Our estimate of 4056 genes in CPCRI-1 and 4286 genes in CPCRI-3 is consistent with this observation. The *Pseudomonas* genus had an average genome size of 6.0 Mb and encoded an average of 5366 protein coding genes (Table S4). Though CPCRI-2 had a genome size of 5.9 Mb, and coded for about 4637 proteins, this number is similar to those observed in *Pseudomonas putida* BIRD-1, a PGPR [10].

**Table 3:** Gene prediction and annotation summary.

	CPCRI-1	CPCRI-2	CPCRI-3
# of predicted protein-coding gene	4,056	4,637	4,286
Average GC content of Protein-coding genes (%)	56.55	64.12	55.4
Average gene length (bp)	972	981	951
# of tRNA genes	74	61	74
Average length of tRNA gene (bp)	76	75	76
# of rRNA genes	5	6	6
# of protein-coding genes with at least one significant BLASTX result	4,053	4,596	4,216
# of BLASTX protein hit present in UniProt	3,747	4,551	3,674
# of protein-coding genes assigned GO term	2,906	3,379	2,515
# of protein-coding genes assigned an InterPro id	3,466	4,097	3,063

The average GC content of the protein coding genes in CPCRI-1 (56.55%), CPCRI-2 (64.12%) and CPCRI-3 (55.4%) and their relation with the genomic GC content were estimated and are presented in Table 3, Table S3, S4. The GC distribution for all three positions in the codon for protein-coding genes (Fig. S2) showed that the average GC content was the highest for the third base position and lowest for second position within the codon. Though the overall trend was similar between the bacteria, the GC content for CPCRI-2 at the third base was ∼85% compared to only 68% for CPCRI-1 and CPCRI-3. The codon usage analysis (Fig. S3) showed that CTG that codes for Leu (L) is the most used codon in all three bacteria.

In addition to the protein coding genes, we predicted 74, 61, and 74 tRNA genes in CPCRI-1, CPCRI-1, and CPCRI-3, respectively (Table S6A, B, C). The predicted genes represented 20–21 different tRNAs corresponding to the universal codons. Our analysis additionally identified 5–6 different rRNA genes (Table 3 & Table S7).

**Figure 1:**
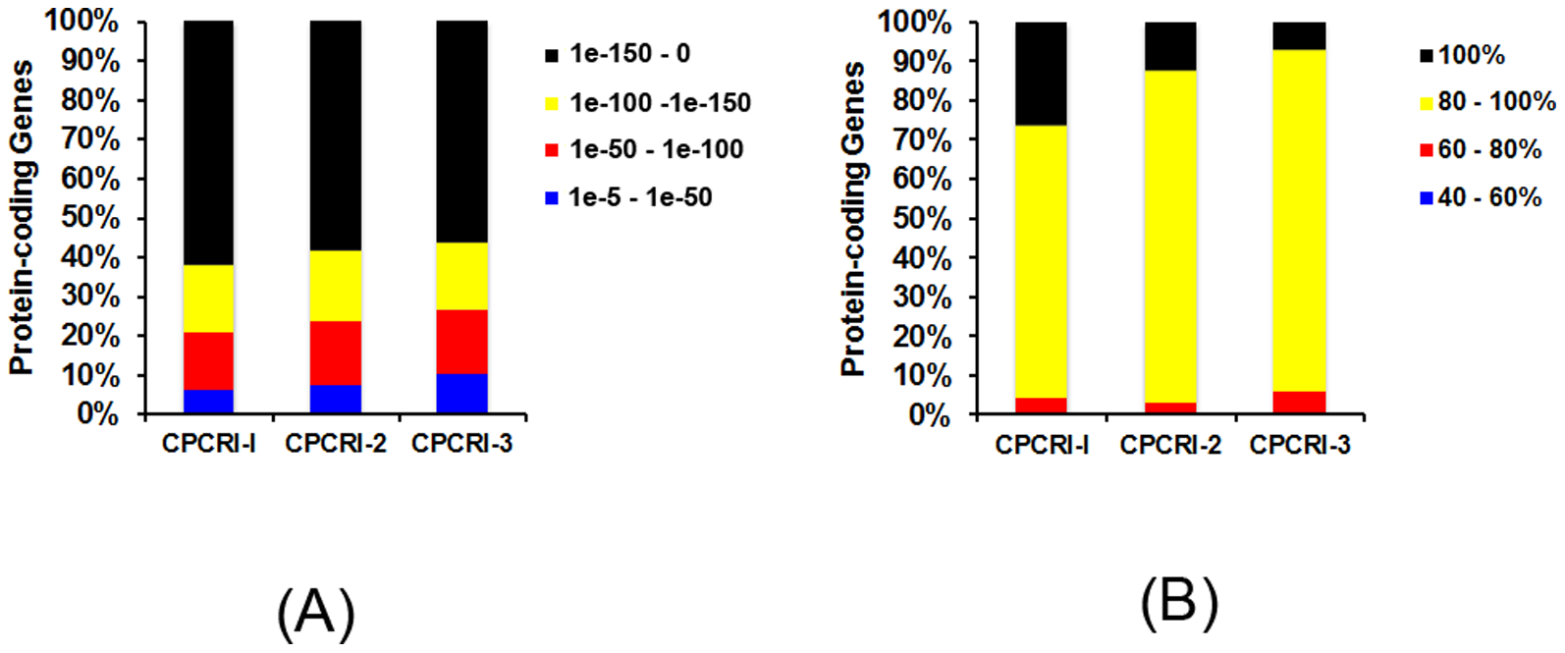
Protein coding genes. Stacked bar graph representing the percentage of predicted protein coding genes with significant matches (E-value< = 1e10^−5^) (A) in the NCBI nr-protein database identified using BLASTX and (B) the proportion of proteins binned by percent identity measured by BLASTX alignment.

To further understand the bacterial strains, the predicted protein-coding genes identified using Glimmer-MG were compared against the non-redundant (nr) NCBI protein database using BLASTX [27]. We found that a majority of the predicted protein-coding genes (98%) had a homologous protein sequence in the NCBI non-redundant (nr) protein database (Fig. 1). Among the genes with homologs, >90% of genes had a high confidence match (E-value< = 1.0 e^−50^) and >93% had identity of at least 80% with a putative homolog (Fig. 1A, B). Interestingly, 31, 59 and 91 genes from CPCRI-1, 2 and 3, respectively, showed no significant identity to sequences in the NCBI database. Of the protein with no significant identity, we annotated protein domains for 15, 21 and 29 genes from CPCRI-1, 2 and 3, respectively, using InterPro [28] and CDD [29]. We further annotated all the protein-coding genes with known protein domains using UniProt database (Table S8A, B, and C) [30].

### Phylogeny analysis

**Figure 2:**
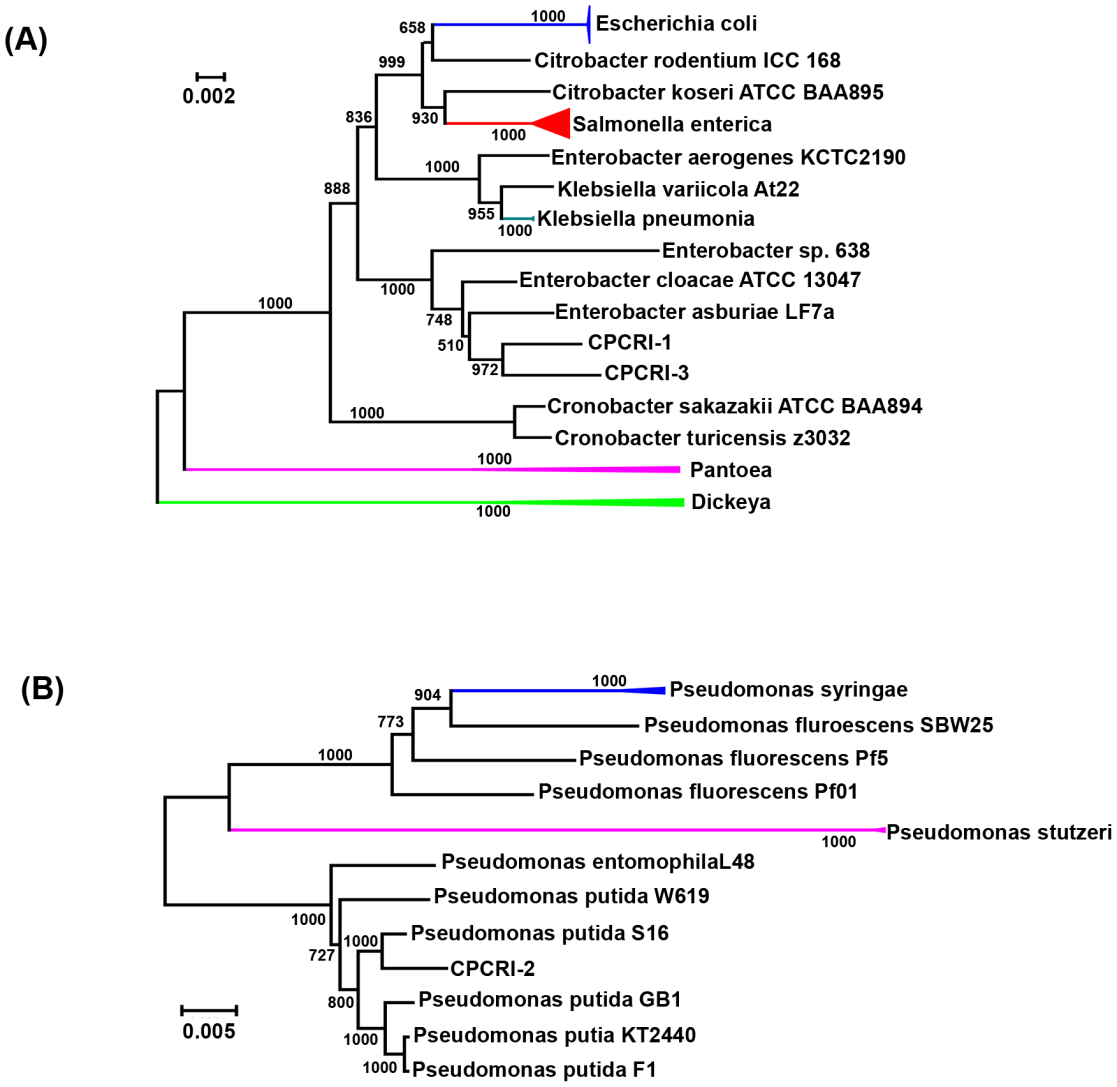
Phylogenetic tree. Using 31 conserved housekeeping protein-coding genes from (A) CPCRI-1 and CPCRI-3, (B) CPCRI-2, a phylogenetic tree was generated using AMPHORA2 [24], [94] and ClustalW [95]. The colored branch/node represents node where multiple strains of the same species are collapsed into a single species for representation.

The phylogenetic analysis performed using a set of 31 conserved housekeeping protein-coding genes [24] revealed that the CPCRI-1 and CPCRI-3 genomes were closely related to the *Enterobacter cloacae* group (Fig. 2A). The CPCRI-2 genome was found to be closely related to the *Pseudomonas putida* group. CPCRI-1 grouped with *Enterobacter asburiae* strain LF7a and *Enterobacter cloacae* ATCC 13047, both of which are members of the *Enterobacter cloacae* complex. CPCRI-3 clustered closely with *Enterobacter asburiae* strain LF7a and *Enterobacter* sp. 638 [31], an endophyte of poplar trees. The closest relative to CPCRI-2 was *Pseudomonas putida* strain S16, a gram-negative soil bacterium with an ability to degrade aromatic and heterocyclic compounds, such as nicotine, benzoate, and phenylalanine [32]. Taxonomy based study using MEGAN4 [33] showed that CPCRI-1 and CPCRI-3 belong to *Enterobacteriacea* family and CPCRI-2 belong to *Pseudomonas* genus (Fig. S4, Result S1). Consistent with this, Biolog analysis indicated CPCRI-2 to be *Pseudomonas putida* [20]. Although Biolog analysis at low confidence level, indicated CPCRI-3 to be *Pantoea agglomerans* [22], a member of the *Enterobacteriaceae*, the genome sequence of CPCRI-1 did not reveal similarity to any sequenced bacterium at the species level.

### Pairwise genome comparison with existing bacterial genomes

**Figure 3:**
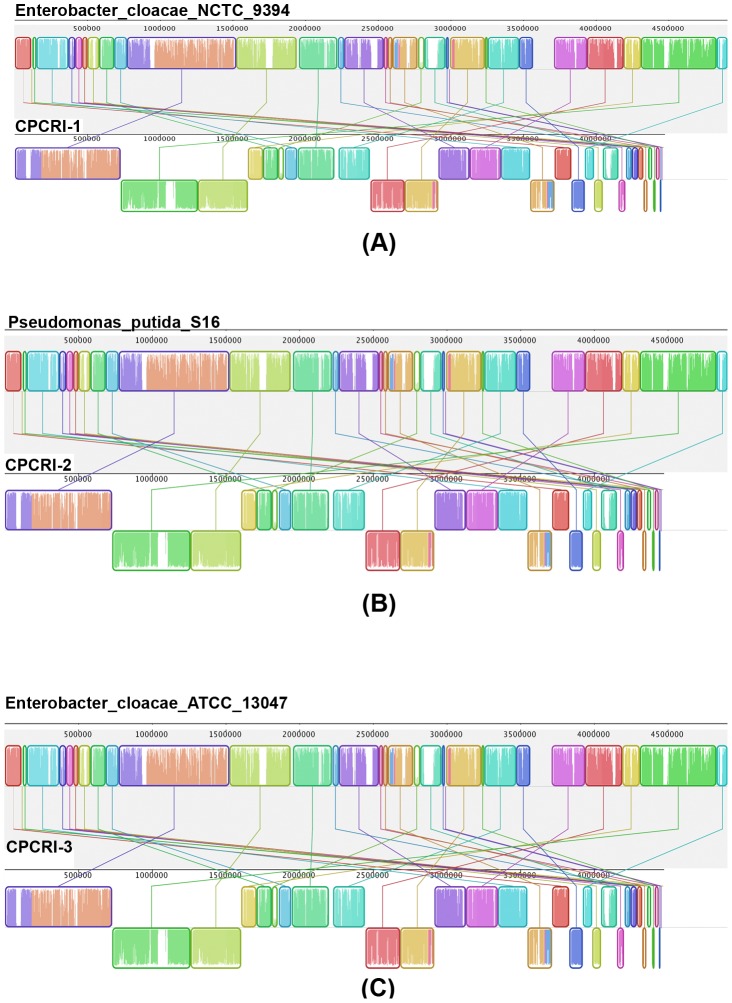
Genome comparison. Pairwise alignment of CPCRI-1, CPCRI-2, CPCRI-3 genome with *Enterobacter cloacae NCTC 9394*, *Pseudomonas putida S16* and *Enterobacter cloacae ATCC 13047*, respectively using the progressive Mauve aligner [34]. The colored blocks represent the homologous region between the genomes that are internally free from genomic rearrangement.

We performed a pairwise genome comparison of our assembled bacterial genomes against 40 different bacteria using progressive Mauve aligner [34]. The bacterial groups identified for analysis included *Enterobacter*, *Escherichia coli*, *Pseudomonas putida*, *Citrobacter*, *Dickeya*, *Klebsiella*, *Pantoea*, *Salmonella*, *Shigella*, *Azotobacter*, *Bradyrhizobium*, *Mesorhizobium* and *Rhizobium*. The genome level comparison showed that CPCRI-1 had the highest similarity to *Enterobacter cloacae NCTC 9394i* (similarity score of 92.69%, coverage of 89.02%). In addition, CPCRI-2 was closest to *Pseudomonas putida S16* (similarity score of 89.88%, coverage of 85.0%), and CPCRI-3 to be most similar to *Enterobacter cloacae ATCC 13047* (similarity score of 81.58%, coverage of 78.56%; Table 4, Table S9 and Fig. 3). These results are consistent with the phylogenetic analysis findings that showed CPCRI-1 and CPCRI-3 belong to *Enterobacter cloacae* group and CPCRI-2 to the *Pseudomonas putida* group.

**Table 4:** Pairwise comparison of CPCRI-1, CPCRI-2, and CPCRI-3 genomes against bacteria genomes using progressive Mauve aligner [34].

Bacteria	CPCRI-1	CPCRI-2	CPCRI-3
*Enterobacter asburiae LF7a*	84.94/83.15	65.25/14.5	81.26/78.61
*Enterobacter cloacae ATCC 13047*	86.20/87.75	65.88/14.67	**81.58/78.56** *
*Enterobacter cloacae EcWSU1*	85.86/86.70	65.38/14.88	81.4/77.95
*Enterobacter cloacae NCTC 9394*	**92.69/89.02** *	65.98/14.47	81.4/77.38
*Enterobacter cloacae SCF1*	79.12/72.00	66.85/12.69	78.3/66.83
*Enterobacter sp. 638*	82.0/78.73	64.79/13.36	80.57/75.66
*Pseudomonas putida BIRD-1*	65.60/18.09	87.84/81.05	65.19/17.06
*Pseudomonas putida F1*	65.50/18.94	88.05/83.70	65.2/17.04
*Pseudomonas putida GB-1*	65.47/18.7	87.7/82.97	65.16/17.57
*Pseudomonas putida KT2440*	65.57/18.14	87.69/83.0	65.21/17.0
*Pseudomonas putida S16*	65.91/18.01	**89.88/85.0** *	65.31/16.91
*Pseudomonas putida W619*	65.50/18.46	84.67/78.64	65.14/16.84
*Pseudomonas aeruginosa LESB58*	65.87/15.85	75.35/59.15	65.56/15.53
*Pseudomonas fluorescens F113*	65.43/18.89	76.3/66.19	64.93/17.05
*Pseudomonas syringae pv tomato str DC3000*	65.26/17.91	74.91/55.95	64.6/16.93

Percentage sequence similarity/genome coverage in the conserved block is shown.

*Bacteria showing highest similarity/genome coverage with sequenced bacteria CPCRI-1, CPCRI-2 and CPCRI-3.

### Functional analysis of the bacterial genome

**Figure 4:**
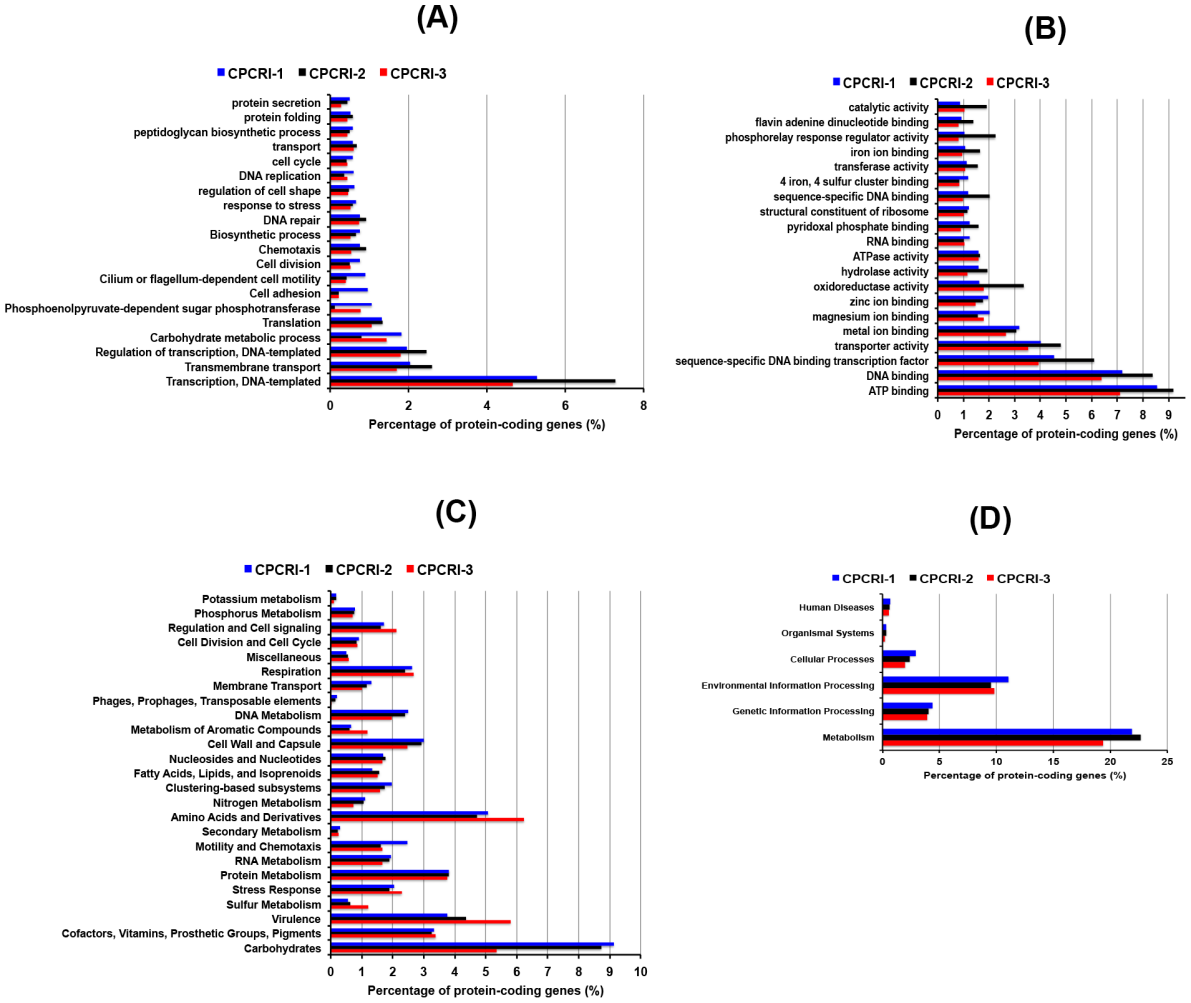
Functional annotation. The percentage of predicted coding genes of CPCRI PGPR genomes into (A) GO biological classes, (B) GO molecular classes, (C) SEED classification, (D) KEGG pathway classification.

We performed functional analysis of the annotated genomes using gene onotology (GO), SEED classification and KEGG pathways. The GO based classification of the genes revealed 2,200 to 3,000 (2,562 for CPCRI-1, 3,020 for CPCRI-2, and 2,226 for CPCRI-3) genes associated with at least one molecular function, 1,500–1,900 (1,836 for CPCRI-1, 1,918 for CPCRI-2, and 1,555 for CPCRI-3) genes associated with at least one biological process, and 1,200–1,500 (1,487 for CPCRI-1, 1,530 for CPCRI-2, and 1,226 for CPCRI-3) genes associated with at least one cellular component (Table S10). The Carbohydrate metabolism, chemotaxis, cell adhesion, cilium or flagellum-dependent related motility, response to stress, iron ion binding, oxidoreductase activity are among the top 20 GO biological processes and molecular functions found in the bacteria (Fig. 4A, B). These terms are related to the functional class of genes that aid the plant growth.

The SEED based classification [35] analysis of the proteins, performed using MEGAN4 [33] assigned functional roles to the annotated genes that were then grouped into one or more subsystems. MEGAN4 classified 1998, 2202 and 2030 annotated genes from CPCRI-1, CPCRI-2 and CPCRI-3, respectively into 25 functional categories (Fig. 4C). A large number of genes fall into carbohydrate metabolism, stress response, motility and chemotaxis and metabolism of aromatic compounds that helps in plant growth. Annotation against KEGG pathway classified the protein-coding genes into six different pathway categories: metabolism, genetic information processing, environmental information processing, cellular processes, organismal systems and human diseases (Fig. 4D). The metabolism and environment information processing category pathways were highly represented in all three bacteria. Overall we found that many genes fall into the functional classes that support plant growth.

### Plant growth promoting properties

In the genomic sequence of three PGPRs sequenced we identified genes that can be attributed to their ability to improve nutrient availability, suppress pathogenic fungi, resist oxidative stress, quorum sensing and ability to break down aromatic and toxic compounds and other abiotic stress (Table 5). The genomes of CPCRI-1, 2 and 3 possessed genes encoding glucose dehydrogenase activity while, CPCRI-1 and 2 carried the co-factor pyrrolo-quinolone quinine (*pqq*) gene cluster which is involved in solubilization of mineral phosphates fixed in soil particles [36]. Indole acetic acid (IAA) is an important hormone that helps in plant growth [37]. Although IAA production was observed in culture from the three PGPRs was low (Table 1), CPCRI-1 and CPCRI-3 genomes contained *ipd*C that codes for indolepyruvate decarboxylase, an enzyme that produces indole acetic acid from tryptophan [38]. In these genomes we also found some of the trp cluster (*trp*A, B, D, C, R) genes involved in tryptophan biosynthesis. These genes may play a role in synthesis of tryptophan used in multiple biological processes, including IAA biosynthesis. The 1-aminocyclopropane-1-carboxylate (ACC) deaminase has been shown in symbiotic bacteria to function in lowering the plant ethylene known to inhibit the nodulation process [39]. We identified in CPCRI-2 *acd*S gene homologue that codes for ACC deaminase enzyme. In CPCRI-1 and 3, we found, *rim*M [37] and *dcy*D [40], both of which also code for ACC deaminase. In addition, we found genes involved in hydrogen sulfide (H_2_S) biosynthesis in all the three PGPR genomes (CPCRI-1, Gene#2608; CPCRI-2, Gene#3152; CPCRI-3, Gene#3465-67, 3470-72). Recently, H_2_S has been reported to increase plant growth and seed germination [41] and the H_2_S production by the PGPRs may play an analogous role in plant roots they colonize.

**Table 5:** List of genes attributable to plant growth promotion traits in the CPCRI PGPR genomes.

Plant growth promotion traits	CPCRI-1	CPCRI-2	CPCRI-3	Genes with potential for conferring PGP traits
Phosphate solubilization	+	+	+	*pqq*, glucose dehydrogenase gene homolog, *pst*A, B, C
IAA production	+	−	+	*ipd*C
Siderophore production	+	+	+	*pvd*, pyoverdine homologous genes, *fpv*A, *mbt*H, *acr*A,B, *fhu*
ACC deaminase activity	+	+	+	*acd*S, *rim*M, *dcy*D
Acetoin & butanediol synthesis	+	+	+	*als*, *bud*A, C, *pox*B
Phenazine production	+	+	+	*phz*F
Chitinase production	+	+	+	Chitinase gene homolog
4-hydroxybenzoate production	+	+	+	*ubi*C
Pyocin	−	+	−	Pyocin gene homolog
Trehalose metabolism	+	+	+	Trehalose synthase gene homolog
H_2_S production	+	+	+	*cys*C, J, I, N
Quorum sensing	+	−	+	*lux*S, *lsr*
Heat shock proteins	+	+	+	*dna*J, K, *gro*E
Cold shock proteins	+	+	+	*csp*A, C, D, E
Glycine-betaine production	+	+	+	*sox*B, *opu*, *pro*X, glycine betaine gene homolog
Peroxidases	+	+	+	*osm*C, glutathione peroxidase genes similar to *Enterobacterasburiae, oxy*R
Catalases	+	+	+	Catalase gene homolog
Superoxide dismutase	+	+	+	*sod*B,C, super oxide dismutase gene homologs
GABA production	+	+	−	*gab*D, T

+ indicates presence of genes.

− indicates absence of genes.

Among recently described volatile molecules known to directly influence the plant growth promotion are acetoin and 2,3-butanediol [42]. The CPCRI genomes encode *bud*C, *bud*A [43] and *als* [44], all of which are involved in the production of acetoin and 2,3-butanediol.

Many PGPRs are known to have biocontrol activities. Production and secretion of siderophores widely used by bacteria for iron acquisition is one of the modes of biocontrol activity. While CPCRI-2 encoded 41 genes involved in the production and utilization of pyoverdine, siderophore, CPCRI-1 and 3, have additional genes such as *fpv* and *mbt* [45] that are linked to pyoverdine production. Besides the genes for pyoverdine, the gene cluster responsible for synthesis of temperature regulated achromobactin siderophore, *acr*A and *acr*B, were also identified in all the three PGPR genomes. In addition to siderophores, chemicals such as phenazine and 4-hydroxybenzoate produced by the PGPRs act as antibiotics and suppress plant pathogenic microbes. In all the three genomes we were able to identify the *phz*F involved in phenazine synthesis and *ubi*C that codes for chorismatelyase involved in 4-hydroxybenzoate synthesis. Also, in CPCRI-2 we identified a homologue of gene associated with the synthesis of anti-microbial compound pyocin [46]. In addition to these, in CPCRI-1 and CPCRI-2 genomes we identified *gab*D and *gab*T involved in production of pest/disease suppressing γ-aminobutyric acid (GABA) [46]. The whole genomes of the three bacteria coded for several genes that encode peroxidases, catalases, superoxide dismutase, and glutathione transferases, all of which alleviate oxidative stress in plants (Fig. S5).

### Carbohydrate metabolism

Analyses of CPCRI-1, CPCRI-2 and CPCRI-3 genomes showed that they carried genes consistent with their ability to survive in soil environment and plant rhizospheres. The genomes of all 3 bacteria encode genes for central carbohydrate metabolism, including the tricarboxylic acid cycle, the Entner-Doudoroff pathway, glycolysis, gluconeogenesis, pyruvate metabolism and the pentose-phosphate pathways. However, the methyl citrate cycle for propionate metabolism (Table S11) was identified only in CPCRI-2. All the three bacteria carried genes for galactose, fructose, mannose, gluconate and glycogen metabolism, however CPCRI-1 and CPCRI-3 genomes showed the presence of a larger number of metabolic pathways for monosaccharides, disaccharides, oligosaccharides, and polysaccharides, than the CPCRI-2 genome. This indicated that CPCRI-1 and CPCRI-3 could use a large variety of plant-derived carbohydrates as carbon source. Additionally, CPCRI-1 and CPCRI-3 encode genes that can support the use of L-rhamnose, L-arabinose, xylose, trehalose, maltose, lactose and β-glucosides as a carbon source, even though utilization of lactose as a sole carbon source is a characteristic of the *Enterobacteriaceae* family [31]. These findings are consistent with the Biolog studies that demonstrated the ability of CPCRI-1 and CPCRI-3 to use L-rhamnose, trehalose, maltose and lactose.

Trehalose, a disaccharide, is accumulated by many microorganisms growing under high salt or osmotic stress and has been shown to play an important role in *Rhizobium*-legume symbiosis [47]. Accumulation of trehalose in *Bradyrhizobium japonicum* enhances its survival under conditions of salinity stress and plays an important role in the development of symbiotic nitrogen-fixing root nodules on soybean plants [48]. We observed that while all the three PGPRs encoded genes that support trehalose biosynthesis, CPCRI-1 and CPCRI-3 also encoded genes for exogenous trehalose uptake that can potential allow them to use exogenous trehalose.

Malonate metabolism has been characterized in various symbiotic bacteria, such as *A. calcoaceticus*, *K. pneumoniae*, *P. fluorescens* and *P. putida* [49], [50]. All three bacterial genomes reported in this study contain genes involved in malonate metabolism. In CPCRI-1 genome, we observed a cluster of nine genes *mdc*ABCDEFGHR (Fig. S6A; CPCRI-1, Gene#3919-3927; CPCRI-3 Gene#2395-2403) involved in malonate decarboxylation (Fig. S6C). Also, CPCRI-2 encodes a nine gene malonate cluster *mdc*MLHGEDCBA (CPCRI-2, Gene#3117-3125; Fig. S6B). This suggests that all the three bacteria are capable of malonate utilization.

### Degradation of aromatic compounds

In addition to the carbohydrate metabolism pathway genes, the CPCRI-2 genome coded for genes involved in the degradation of various aromatic compounds such as benzoate, 2,4-dichlorobenzoate, 1,2-dichloroethane, tetrachloroethane and bisphenolA. The β-ketoadipate pathway, an important bacterial energy source, has been identified in the *Pseudomonas* species and many members of *Rhizobiaceae* family of soil microorganisms [51], [52]. The CPCRI-2 genome contains β-ketoadipate pathway genes involved in degradation of lignin derived aromatic compounds. Further in CPCRI-2, we also found genes involved in the metabolism of polyhydroxybutyrate (PHB), an aliphatic polyester synthesized by several bacteria as a means of carbon storage and a source of reducing equivalents in starving conditions [53]. PHB is stored intracellularly as granules and improves bacterial tolerance to high temperatures, H_2_O_2_ exposure, UV-irradiation, desiccation, and osmotic stress [53], [54]. Interestingly, the three genomes also encoded *ars*C gene [45] which may play a role in detoxifying arsenic.

### Quorum sensing

Both CPCRI-1 and CPCRI-3 encoded the autoinducer-2 (AI-2) gene [*lux*S; Gene #3755 (CPCRI-1); Gene #3423 (CPCRI-3)]. AI-2 is a small molecule produced by a number of bacterial species, implicated in the regulation of biofilm formation, motility and production of virulence factors [55]. AI-2 has been suggested to act directly through quorum sensing or indirectly through modulation of cellular metabolism. AI-2 dependent quorum sensing system has been demonstrated to be crucial for symbiosis between *Sinorhizobium meliloti* and legumes. *S. meliloti* can respond to the AI-2 signal by up-regulating transcription of its *lsr*-like operon [56]. Genomes of CPCRI-1 and CPCRI-3 also code for *lsr* operon, which contains genes encoding the transport apparatus responsible for internalizing, phosphorylating and processing of the AI-2 signal. The *lsr* operon comprises of six genes, *lsr*ACDBFG [57] is present in CPCRI-1 (Gene#2142-2147) and CPCRI-3 (Gene#4093-4098) genomes. The *lsr*B encodes the ligand binding protein, *lsr*C and *lsr*D each encode a transmembrane protein, and *lsr*A encodes a cytoplasmic protein responsible for ATP hydrolysis during transport. In addition to AI-2 quorum systems, non-lux based quorum sensing proteins controlled by *rib*B gene [58] was found in the genomes of all the three CPCRI bacteria (CPCRI-1: Gene#2104, CPCRI-2: Gene#1422, Gene#1433 and for CPCRI-3: Gene#3641). Gene *rib*B is a homolog of the *Escherichia coli* gene for 3,4-dihydroxy-2-butanone 4-phosphate synthase, a key enzyme for riboflavin synthesis, which along with *qsr*P, *acf*A, *qsr*V, and *qsr*7 have been proved as non-lux based QSR protein producing genes [58]. In CPCRI-2 (*Pseudomonas putida*) genome, Lux-R system (Gene#3078, 3458, 3799, 3918, 4032, 4115, 4123, 4282, 4600 and 4637) that is involved in acyl-homoserine lactone (ACL) controlled quorum sensing system was found [59]. A collection of genes such as *gac*A (Gene#4600), *rsmA* (Gene#3492) and *rpo*S (Gene#3005) that are known to regulate and network LasRI and RhlRI quorum sensing systems in *Pseudomonas aeruginosa* [60] was also found in CPCRI-2. These observations suggest that CPCRI-1, 2 and 3 may have quorum sensing ability that can contribute to their symbiotic relationship with the host plant.

### PGPR fitness conferring genes

Production of heat-shock proteins, cold-shock proteins and osmoregulants in the bacteria regulate survival under harsh conditions. The genomes of all the three CPCRI isolates carried heat-shock protein genes like *dna*J, K and *gro*E, cold-shock proteins genes such as *csp*A, C, D, and E, and several copies of osmoprotectant glycine betaine synthesis genes. Other genes, *gac*S [61], *sox*S, R, *oxy*R [62] involved in protecting plants against oxidative stress were also found in the CPCRI genomes. We found *xer*C gene [63] in all the three CPCRI genomes. The *xer*C gene product, a site recombinase, is critical for the PGPRs to be an effective rhizosphere colonizer [63].

### Comparison with non-PGPR bacteria

We compared the genes present in CPCRI bacteria with non-PGPR bacteria of similar strain. The CPCRI-1, 3 genomes were compared with *Enterobacter cloacae EcWSU1* [64] and *Enterobacter cloacae subsp. cloacae ATCC 13047* [65], whereas CPCRI-2 genome was compared with *Pseudomonas putida strain S16* [32]. Comparison was performed at functional classification level using GO, SEED and KEGG annotation (Table S12A–I).

Comparison of CPCRI-1, 3 with non-PGPR showed that pyrroloquinoline quinone (*pqq*) biosynthetic gene which is involved in solubilization of mineral phosphates was only present in CPCRI-1, 3 genomes. The acetoin-production gene, which is associated with butanediol dehydrogenase activity, was absent in non-PGPRs. The iron-scavenging group of genes involved in siderophore synthesis and their uptake was more enriched in CPCRI-1, 3 as compared to non-PGPRs. Also, CPCRI-1 genome showed adhesion group of genes to be highly enriched as compared to non-PGPRs.

Comparison of CPCRI-2 with non-PGPR revealed many functional groups that included some key plant growth traits. The widespread colonization island, siderophore enterobactin, pyrroloquinoline quinone (*pqq*) biosynthetic and phenazine (*phz*) biosynthesis genes present in CPCRI-2 were completely absent in *Pseudomonas putida strain S16*. Genes related to adhesion, iron scavenging and sulfur metabolism were more enriched in CPCRI-2 as compared to *Pseudomonas putida strain S16*.

## Discussion

In this study we reported the whole genome sequencing and analysis of three PGPRs, CPCRI-1, CPCRI-2 and CPCRI-3 isolated from coconut [16], cocoa [20] and arecanut [22], respectively. The genomic level characterization reported here of PGPRs, to our knowledge is the first for rhizobacteria isolated from coconut, cocoa and arecanut. Usually the bacterial genomes are compact and tightly packed with genes and other functional elements and range in size from 0.5 to 10 Mb, with coding regions averaging ∼1 Kb [66]. Following assembly we estimated the genome sizes to be 4.7 Mb for CPCRI-1, 5.9 Mb for CPCRI-2 and 5.1 Mb for CPCRI-3 and there was a good correlation observed between the genome size and genome numbers of the three PGPRs as earlier reported in other studies [26]. The genome size of our *Enterobacter* spp. (CPCRI-1 and 3) was comparable to those of the others isolated from plantation crops such as sugar cane [67] and poplar [31], which had 4.9 and 4.6 Mb sizes respectively. Similarly, the genome size of *Pseudomonas* from cocoa (CPCRI-2) matched with that of the *Pseudomonas aurantiaca* obtained from sugar cane [68]. The GC contents recorded for CPCRI-1 and 3 matched well within the range reported for *Enterobacteriaceae* (38–60%) family and the range reported for the genus *Enterobacter* (52–60%) [69]. Similarly, GC content of CPCRI-2 was observed to fit well in the range expected for *Pseudomonas* genus (58–69%) [70]. Earlier studies have revealed that the GC content of the total genome usually matched with GC content of protein coding genes, spacer genes and stable RNA genes [71]. We could also observe a strong positive correlation between the GC content of the protein coding genes with GC content of total genome of CPCRI-1, 2 and 3 (Table 3, Table S3 and S4). Another interesting observation was about the codon usage pattern: CTG that codes for Leucine (leu) (Fig. S2) was found to be the most preferred codon in the CPCRI isolates as reported earlier in *Escherichia coli* and *Drosophila melanogaster* [72]–[74].

We identified between 4000 and 4600 protein coding genes in each of the three genomes. While a majority of the genes had homologs in the published sequence database, for 31, 59 and 91 proteins in CPCRI-1, CPCRI-2 and CPCRI-3, respectively, no homologs were found, suggesting that these may have novel functions.

Phylogenetic analysis indicated that both the bacteria isolated from coconut and arecanut belonged to *Enterobacteriaceae* and may reflect the fact that both plantation crops belong to the *Arecaceae* family and have similar root niche/environment. The cocoa isolate belonged to the *Pseudomonadaceae* family.

Consistent with the PGP properties we found several genes that function in mineral phosphate solubilization, ACC deaminase function, IAA, acetoin and butanediol production. Previously, genes with similar functions in other PGPRs have been reported [36], [40], [42], [75]–[77]. The genome sequence of *Enterobacters* spp. of coconut and arecanut and *Pseudomonas* from cocoa possessed many genes that have been reported in PGPR isolated from the plantation crops such as poplar and sugarcane. For example, *sod*B, C controlling the superoxide dismutase activity in CPCRI-1 and 3, *oxy*R gene known to regulate production of anti-microbial compound 4-hydroxybenzoate in CPCRI-1, mobility genes *flg*, *flh*, *fim*, and *fli*, in CPCRI-3 had orthologs in *Enterobacter* sp. 638 PGPR isolated from poplar [31]. Similarly, phosphate transporter genes *pst*A, B and C found in CPCRI-1 and 3 had orthologs in the *Enterobacter* spp. SP1 PGPR isolated from sugarcane [67]. Additionally, comparison of CPCRI-1, 2 and 3 genomes against non-PGPR genomes of the same genus showed several plant growth related group of genes that were either absent, like the pyrroloquinoline quinone (*pqq*) biosynthetic process gene, or less enriched in non-PGPR genomes.

In addition to growth promoting functions, PGPRs also indirectly support plant growth by suppressing pathogens [2]. In the PGPR genomes reported in this study, we identified several genes that are known to support the production of antimicrobial compounds such as siderophores, phenazine, 4-hydroxybenzoate and GABA [46]. They also contained genes for chitinase enzyme that can dissolve cell walls of pathogenic fungi, nematodes and insect pests [46]. In addition, CPCRI-2 genome encoded a gene for production of pyocin, a compound that suppresses growth of other related species. The three PGPR genomes also encoded enzymes such as peroxidases, catalases, super oxide dismutases and glutathione transferases all of which are involved in the management of oxidative stresses in plants.

Sulfur is an essential nutrient for plant growth and development and is associated with stress tolerance in plants [78]. Crop plants generally rely on the soil for their sulfur requirement and the mobilization of this sulfur for assimilation by plants is mediated by the microbial community in the soil and rhizosphere [79]. Sulfur-deficient conditions can cause severe losses in crop yield [80]. Sulfur nutrition is demonstrated to be critical in cocoa somatic embryogenesis [81]. In cocoa, elemental sulfur was identified in the xylem of resistant genotypes after infection by the vascular fungal pathogen *Verticillium dahlia* [82]. We found genes involved in H_2_S biosynthesis in all the three PGPRs sequenced and they may, in particular in cocoa PGPR (CPCRI-2), be an important source of sulfur. We have also identified protein coding genes in the three bacteria known to be involved in resistance to copper, cobalt, zinc, arsenic, mercury and cadmium, suggesting that they function in detoxification of these metals.

Sequence analysis also showed that all the three CPCRI isolates have complete gene clusters corresponding to Type II, VI, Sec and Twin arginine targeting gene complexes (Table S13). Some of the past studies have shown that the Type I–VI and Sec secretion systems in rhizobacteria *Pseudomonas fluorescens* and *Variovorax paradoxus* function in promoting plant growth [45], [46], [83], [84]. The presence of these secretion systems in PGPRs may play a role in their plant growth promoting functions and also provide support for their rhizosphere colonization ability [85], [86].

Among the many biological properties of CPCRI isolates, their ability to utilize different carbohydrate sources and survive and grow under a wide range of pH, NaCl concentrations, and temperature would able to help them establish well under changing soil conditions. Accumulation of disaccharide trehalose has been implicated in survival of some of the plant-beneficial symbiotic microorganisms under salt or osmotic stress conditions [47], [48]. We observed that while all the three bacteria are capable of trehalose biosynthesis, CPCRI-1 and CPCRI-3 also have genes (*tre*Y, Z) that will support exogenous trehalose uptake, further indicating that they are capable of tolerating high salinity or osmotic stress. Presence of genes that regulate the production of heat-shock, cold-shock proteins and osmoregulants in CPCRI PGPRs indicate that they have the capabilities to adapt to harsh conditions for their survival.

The genomic information obtained support the observed traits making them ideal candidates for further development as biofertilizers. The genes identified in our draft genome can now be studied for specific functions using knockout strategies. Experiments can be designed to identify the genes involved in the plant colonization and plant growth promotion process. Genetic engineering can be used to further improve the plant growth promoting properties of these bacteria. These findings will help in designing comprehensive strategies for development and use of such PGPRs to support sustainable plantation crop cultivation.

## Materials and Methods

### PGPR strains

About 1512 morphologically distinct heterotrophic bacteria were isolated from coconut, cocoa and arecanut rhizosphere soil samples [13], [15], [18], [22] collected from privately owned farms with the permission of the owner. The different agro-ecological zones in India from which the samples were collected are listed in Table S1. The bacteria were first screened *in vitro* for a dozen important plant growth promoting properties and then tested for growth promotion in rice (for coconut and arecanut isolates) and cowpea (for cocoa isolates). Also, they were tested for growth promotion activity in coconut, cocoa and arecanut seedlings [13], [16], [18], [19]. Based on their plant growth promotion characteristics, three PGPRs, designated here as CPCRI-1 (from coconut), CPCRI-2 (from cocoa) and CPCRI-3 (from arecanut) were given bio labels as RNF267 [16], KGSF20 [20], and KtRA5-88 [22], respectively, based on place/source of isolation and were selected for whole genome sequencing studies.

Bacterial cell morphology was assessed microscopically. Gram's staining was also performed. PGPR identification was done by conventional biochemical assays and Biolog analysis [18], [19]. Cultures grown for 24 h on Biolog universal growth (BUG) agar were collected and processed according to the manufacturer's instructions (Hayward, CA). Briefly, cultures were transferred to inoculating fluid A (IF-A) and inoculum density was adjusted to 98% T using Biolog turbidimeter (Hayward, CA). Using multi-channel pipette, cell suspension was inoculated into Biolog Gen III Microplates (100 µl/well) containing 96 wells that provides 94 phenotypic tests. Plates were incubated at 33°C for 24 h. The optical density at 590 nm produced from the reduction of tetrazolium violet in each well was read after 24 h using a Biolog Microplate reader (version 5.1.1). Identification was performed by comparing the pattern formed in culture wells with possible patterns in the Microstation/MicrologVersion 5.1.1 database. A species identification of the PGPRs isolated coconut, cocoa and arecanut was acknowledged when the similarity index (SIM) and distance (DIS) values were >0.5 and <5.0, respectively [13], [15], [16], [19], [20].

### Genomic DNA isolation

The three PGPRs, CPCRI-1, CPCRI-2 and CPCRI-3, chosen based on their plant beneficial attributes towards coconut, cocoa and arecanut were grown in Tryptic Soy Broth (TSB) medium at 30°C for 24–48 h. Genomic DNA was extracted using Gen Elute bacterial genomic DNA kit (Sigma, USA) as per the manufacturer's instructions. The extracted DNA was resolved on 0.8% agarose gel to check its integrity. The quality of the genomic DNA samples was assessed using Bioanalyzer DNA 7500 chip (Agilent, CA). The DNA yield was estimated on a TBS-380 Mini-Fluorometer (Turner BioSystems, CA) using PicoGreen dsDNA Quantitation Reagent (Molecular Probes, OR)

### Library preparation and multiplexed whole genome shotgun sequencing

Whole genome shotgun libraries were generated from 1 µg genomic DNA using the GS FLX Titanium Rapid Library Preparation Kit (Roche Applied Science, CA) according to the manufacturer's protocol. Rapid library MID Adaptors MID10, MID11 and MID12 (Roche Applied Science, CA) were ligated to the CPCRI-1, CPCRI-2 and CPCRI-3 libraries, respectively. The quality of the libraries (library size ∼1,600 bp) was assessed using Bioanalyzer High sensitivity DNA chip (Agilent, CA). The libraries were titrated by emulsion titrations and based on the percent enrichment, appropriate amount of the libraries were used to set up the large volume emulsion PCRs for each of the individual libraries. The beads containing clonally amplified DNA were enriched and the sequencing primer was annealed. Finally, half of the beads containing the CPCRI-2 libraries were mixed with CPCRI-1 library beads and the other half with the CPCRI-3 library beads. Each set of bead mix was then loaded on a picoTiter plate (half the plate) and sequenced using the GS FLX Titanium Sequencing Kit XL+ (Roche Applied Science, CA). Upon sequencing and processing of the raw data, demultiplexed data were assembled using GS de novo assembler version 2.6 (Roche Applied Science, CA).

### Gene prediction

The protein-coding genes prediction was performed using Glimmer-MG [25], a metagenomics gene prediction program that uses interpolated Markov models (IMMs) to identify the protein-coding regions in the genome. The default setting for Glimmer-MG was used for gene prediction. The tRNA genes in the genome were identified using tRNA-SE program [87]. The BLASTN program (E-value< = 1.0 e^−10^) at WebMGA was used for predicting ribosomal RNA genes [88].

### Gene annotation

For gene annotation, we first compared predicted protein-coding genes against the non-redundant (nr) NCBI protein database using BLASTX (E-value< = 1.0e^−5^) program [27], [89]. BLASTX result was parsed and the top hit database accession numbers were extracted. The accession numbers were then compared against UniProt knowledgebase for annotating genes [90]. The BLASTX result was imported into MEGAN4 [33], [91] to perform KEGG pathway analysis [92] and SEED classification [93] of the proteins. The annotated genes were inspected for identifying those involved in PGP functions, pathogen suppression, abiotic stress tolerance, rhizosphere competence, carbohydrate metabolism and other important relevant functions.

### Phylogenetic analysis

Phylogenetic analysis was performed using AMPHORA2 [24], [94], a phylogenomic inference tool used for genomic phylotyping of bacteria and archaeal genomes. It scans the genome for 31 marker genes, which are universally distributed in both phyla. The 31 marker genes identified in CPCRI-1, CPCRI-2, and CPCRI-3 genome were then aligned using ClustalW [95]. Phylogenetic tree was inferred using bootstrap method available in ClustalW package.

### Genome comparison

We compared our assembled bacterial genomes with the available complete bacterial genomes using progressive Mauve aligner [34] using default settings. The published genomes used in the alignment were obtained from PATRIC database (http://patricbrc.vbi.vt.edu) [96]. The sequence alignment file generated by the aligner was parsed to calculate pairwise similarity. Briefly, we first extracted the conserved blocks from the alignment file and then regions with <50 continuous gaps were considered for computing similarity score based on a pairwise sequence similarity percentage and coverage score which represents the percentage of genome that could be aligned pairwise.

## Supporting Information

Figure S1 CPCRI genomes. Circos plot representing the CPCRI-1 (A), CPCRI-2 (B) and CPCRI-3 (C) genomes. The innermost circle represents the GC content, the second circle from the innermost circle represent non-coding genes, the third circle from inside represents coding genes on negative strand, the fourth circle represents coding genes on positive strand, and the outermost circle represent contigs.
(TIF)

Figure S2 GC-content based on codon position. GC-content distribution at each of the three codon position dervied from proportion of genes with a given GC-content at that position is shown for CPCRI-1 (A) CPCRI-2 (B) and CPCRI-3 (C).
(TIF)

Figure S3 Codon usage. The proportion of each codon (%) used in the CPCRI PGPR genomes computed from the protein-coding genes.
(TIF)

Figure S4 Protein taxonomy tree. Proteins encoded by the CPCRI PGPR genomes analyzed using MEGAN4 [33]. The numbers in bracket represent total number of gene assigned based on MEGAN4 annotation. The number in the bracket correspond to CPCRI-1, CPCRI-2 and CPCRI-3 in that order.
(TIFF)

Figure S5 Number of genes coding for oxidative stress response enzymes in each of the indicated CPCRI PGPR strains.
(TIF)

Figure S6 Malonate gene cluster in (A) CPCRI-1, (B) CPCRI-2, (C) CPCRI-3 genome.
(TIFF)

Table S1 Isolation details of PGPR isolates.
(XLSX)

Table S2 Biological properties of PGPRs (CPCRI-1, CPCRI-2, CPCRI-3).
(XLSX)

Table S3 Genomic properties of *Enterobacteriaceae* family.
(XLSX)

Table S4 Genomic properties of *Pseudomonadeceae* family.
(XLSX)

Table S5 **A.** Predicted protein-coding genes in CPCRI-1. **B.** Predicted protein-coding genes in CPCRI-2. **C.** Predicted protein-coding genes in CPCRI-3.
(XLSX)

Table S6 **A.** Predicted tRNA genes in CPCRI-1 genome. **B.** Predicted tRNA genes in CPCRI-2 genome. **C.** Predicted tRNA genes in CPCRI-3 genome.
(XLSX)

Table S7 Predicted rRNA genes in CPCRI-1, CPCRI-2, CPCRI-3 genome.
(XLSX)

Table S8 **A.** CPCRI-1 protein coding genes annotation. **B.** CPCRI-2 protein coding genes annotation. **C.** CPCRI-3 protein coding genes annotation.
(XLSX)

Table S9 Pairwise comparison of CPCRI-1, CPCRI-2, and CPCRI-3 PGPR genomes against bacteria genomes using progressive Mauve aligner. Percentage sequence similarity/genome coverage in the conserved block is shown.
(XLSX)

Table S10 List of GO terms identified in the bacterial genomes.
(XLSX)

Table S11 Propionate metabolism pathway genes in CPCRI-1, CPCRI-2 and CPCRI-3 PGPR genomes.
(XLSX)

Table S12 **A.** SEED comparison summary for CPCRI-1, 3 vs non-PGPRs (*Enterobacter cloacae EcWSU1, Enterobacter cloacae subsp. cloacae ATCC 13047*). **B.** KEGG comparison summary for CPCRI-1, 3 vs non-PGPRs (*Enterobacter cloacae EcWSU1, Enterobacter cloacae subsp. cloacae ATCC 13047*). **C.** SEED comparison summary for CPCRI-2 vs non-PGPRs (*Pseudomonas putida S16*). **D.** KEGG comparison summary for CPCRI-2 vs non-PGPRs (*Pseudomonas putida S16*) **E.** GO comparison summary of CPCRI-1 vs *Enterobacter cloacae EcWSU1* (a non-PGPR). **F.** GO comparison summary of CPCRI-1 vs *Enterobacter cloacae subsp. cloacae ATCC 13047* (a non-PGPR). **G.** GO comparison summary of CPCRI-2 vs *Pseudomonas putida strain S16* (a non-PGPR). **H.** GO comparison summary of CPCRI-3 vs *Enterobacter cloacae EcWSU1* (a non-PGPR). **I.** GO comparison summary of CPCRI-3 vs *Enterobacter cloacae subsp. cloacae ATCC 13047* (a non-PGPR).
(XLSX)

Table S13 Bacteria secretion system KEGG pathway gene in CPCRI-1, CPCRI-2 and CPCRI-3 genomes.
(XLSX)

Result S1 Protein taxonomy results using MEGAN4 program.
(DOCX)
