# Supplementary figures and images for: Whole Genome Sequencing and Analysis of Plant Growth Promoting Bacteria Isolated from the Rhizosphere of Plantation Crops Coconut, Cocoa and Arecanut
Source: PLoS One. 2014 Aug 27;9(8):e104259. doi: 10.1371/journal.pone.0104259 (PMC4146471; doi:10.1371/journal.pone.0104259)

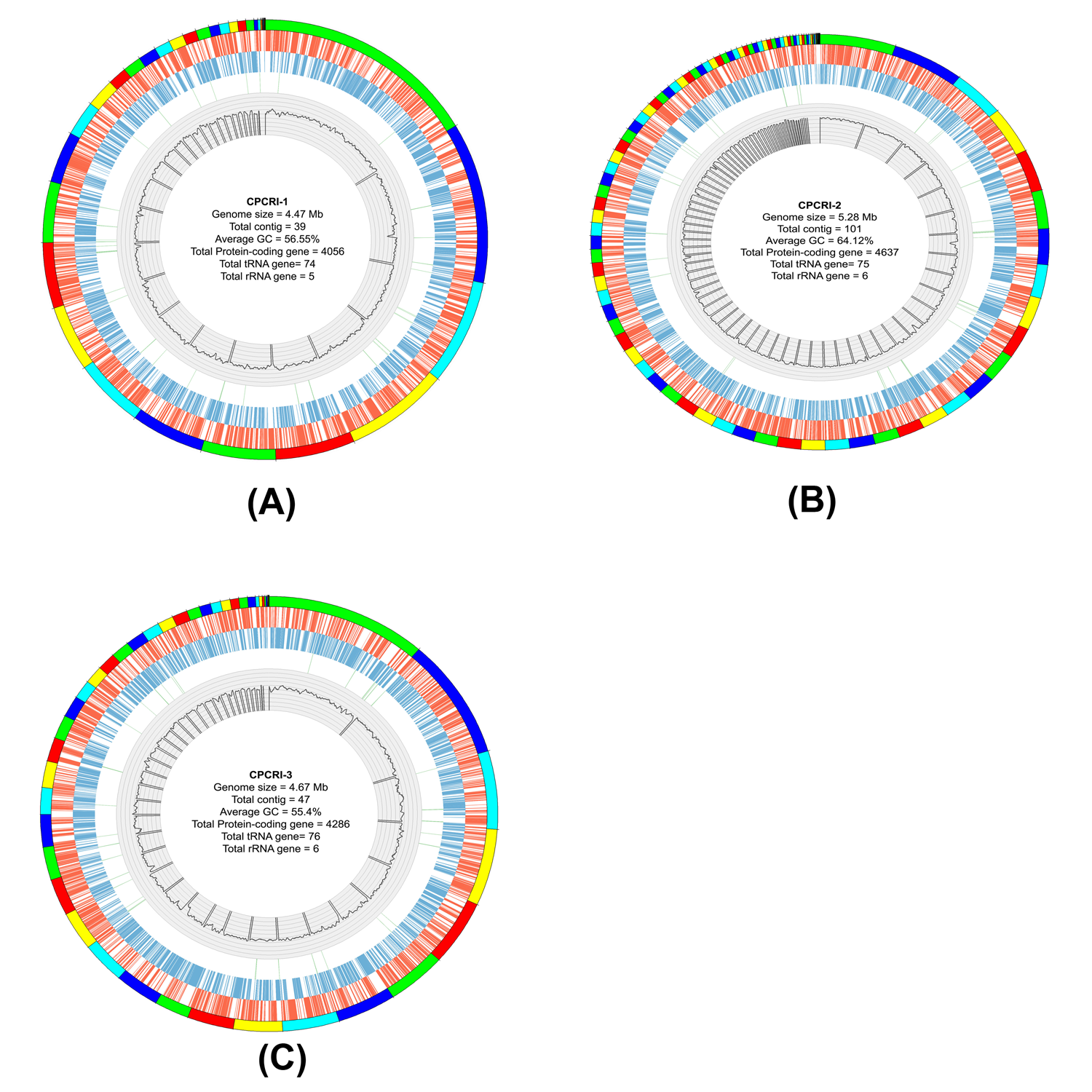

Supplement: Figure S1 — CPCRI genomes. Circos plot representing the CPCRI-1 (A), CPCRI-2 (B) and CPCRI-3 (C) genomes. The innermost circle represents the GC content, the second circle from the innermost circle represent non-coding genes, the third circle from inside represents coding genes on negative strand, the fourth circle represents coding genes on positive strand, and the outermost circle represent contigs. (TIF) [file pone.0104259.s001.tif]

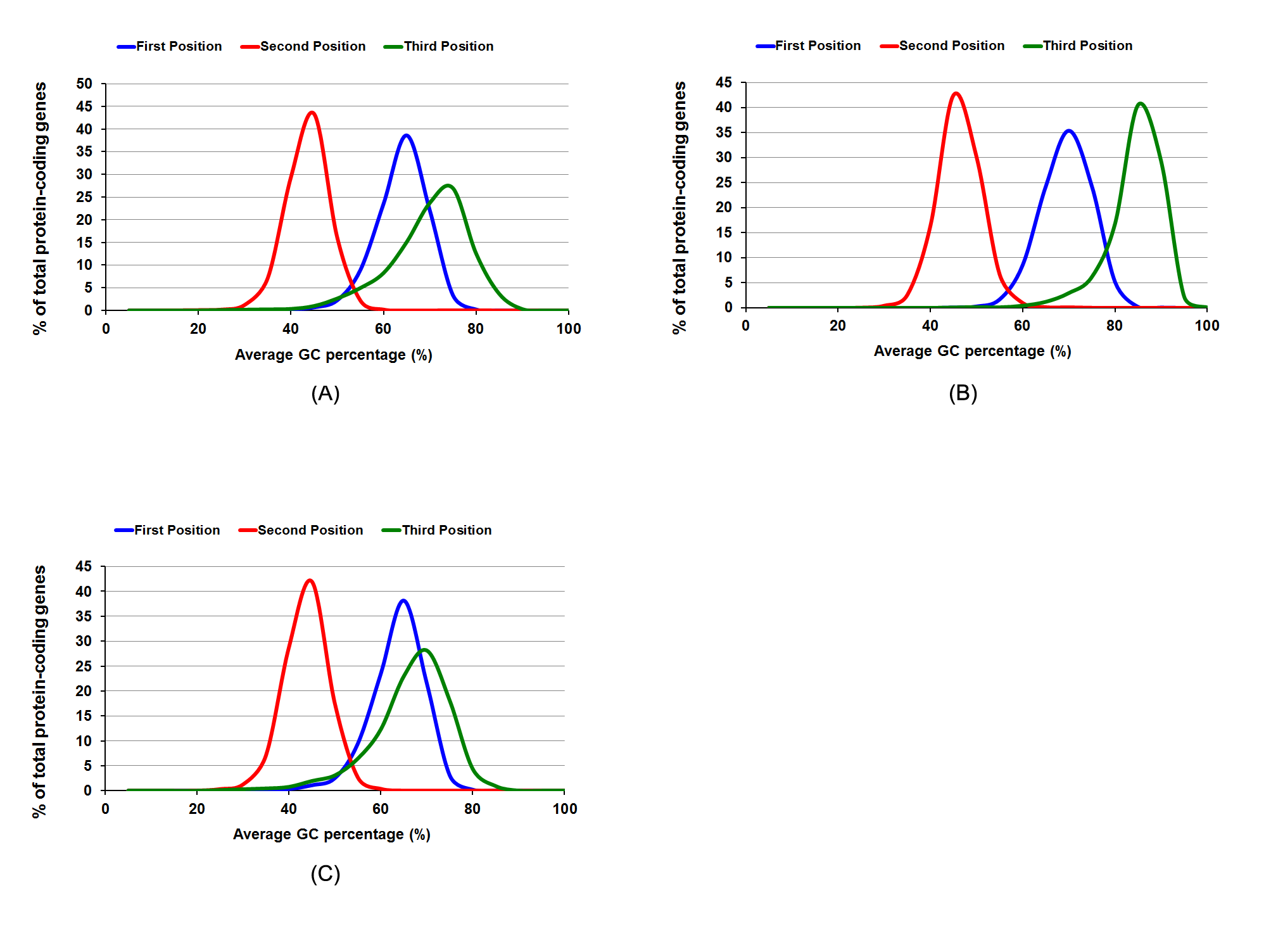

Supplement: Figure S2 — GC-content based on codon position. GC-content distribution at each of the three codon position dervied from proportion of genes with a given GC-content at that position is shown for CPCRI-1 (A) CPCRI-2 (B) and CPCRI-3 (C). (TIF) [file pone.0104259.s002.tif]

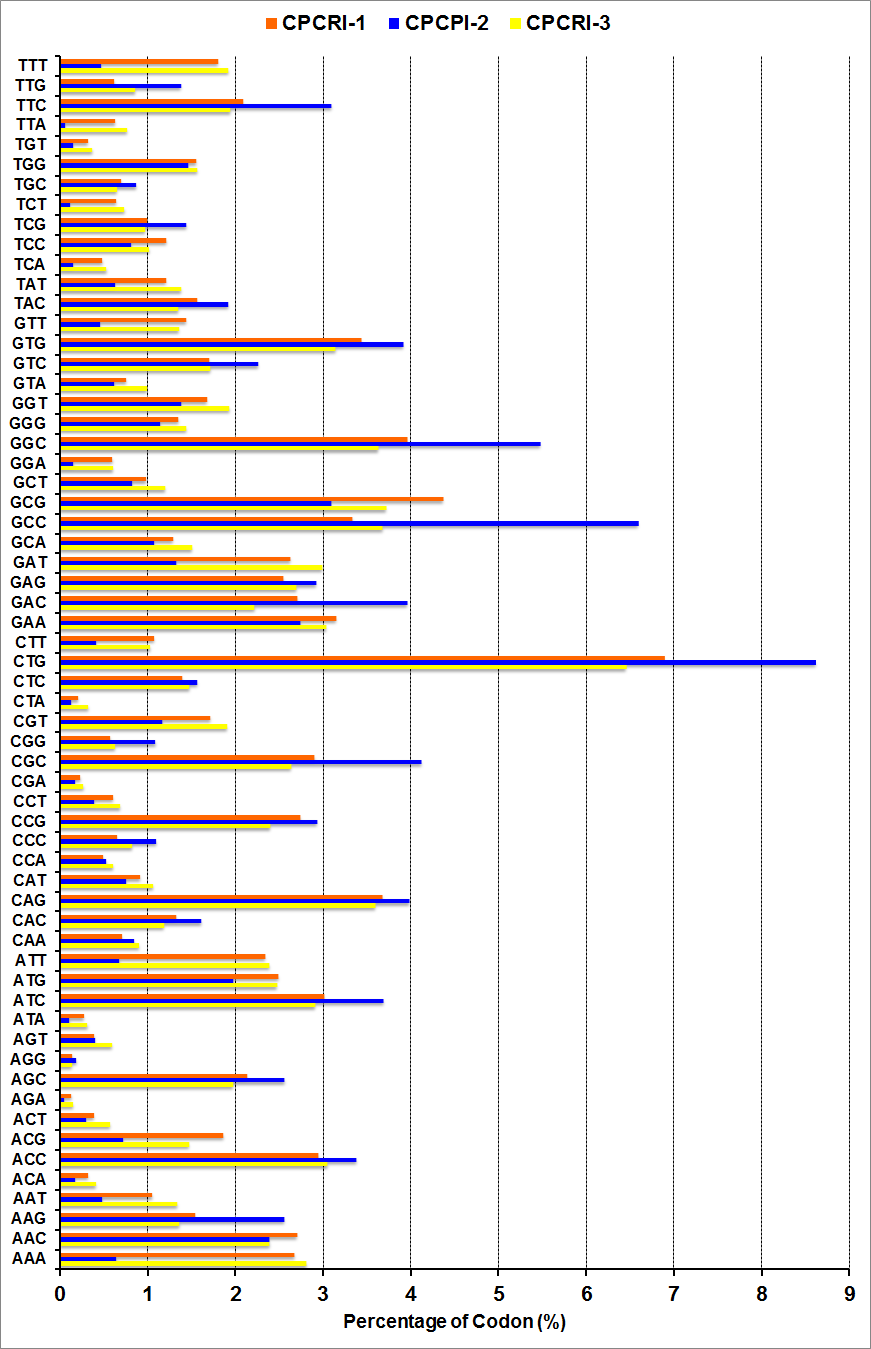

Supplement: Figure S3 — Codon usage. The proportion of each codon (%) used in the CPCRI PGPR genomes computed from the protein-coding genes. (TIF) [file pone.0104259.s003.tif]

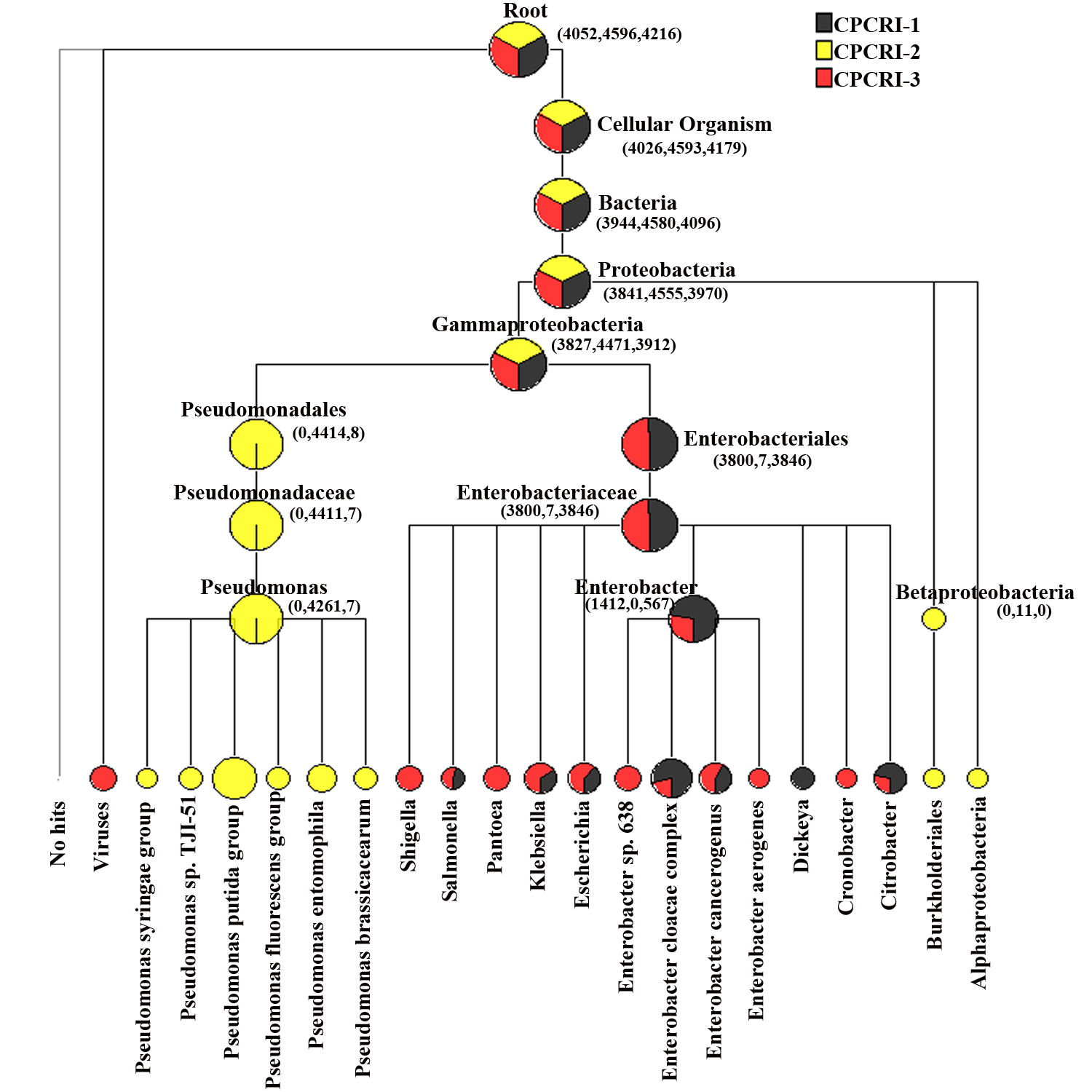

Supplement: Figure S4 — Protein taxonomy tree. Proteins encoded by the CPCRI PGPR genomes analyzed using MEGAN4 [33]. The numbers in bracket represent total number of gene assigned based on MEGAN4 annotation. The number in the bracket correspond to CPCRI-1, CPCRI-2 and CPCRI-3 in that order. (TIFF) [file pone.0104259.s004.tiff]

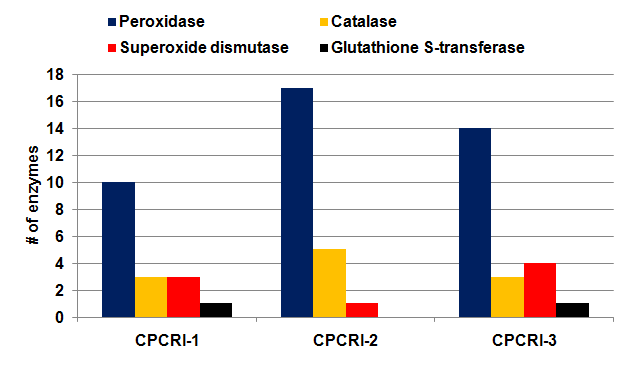

Supplement: Figure S5 — Number of genes coding for oxidative stress response enzymes in each of the indicated CPCRI PGPR strains. (TIF) [file pone.0104259.s005.tif]

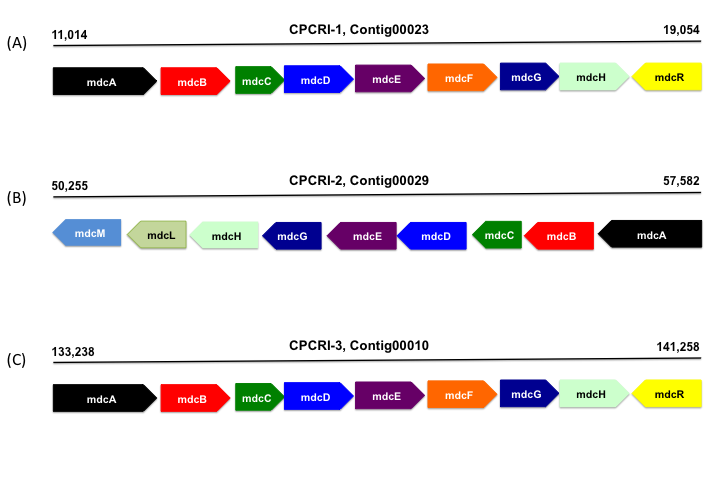

Supplement: Figure S6 — Malonate gene cluster in (A) CPCRI-1, (B) CPCRI-2, (C) CPCRI-3 genome. (TIFF) [file pone.0104259.s006.tiff]
