# Supplementary material for: Whole Genome Sequencing and Analysis of Plant Growth Promoting Bacteria Isolated from the Rhizosphere of Plantation Crops Coconut, Cocoa and Arecanut
Source: PLoS One. 2014 Aug 27;9(8):e104259. doi: 10.1371/journal.pone.0104259 (PMC4146471; doi:10.1371/journal.pone.0104259)
Supplement: Result S1 — Protein taxonomy results using MEGAN4 program. (DOCX) [file pone.0104259.s020.docx]

**Supplementary Results**

Protein Taxonomy

Protein taxonomy analysis using MEGAN4 revealed that all the three CPCRI bacteria belonged to *Gammaproteobacteria* class. It further confirmed that CPCRI-1 and CPCRI-3 belonged to the *Enterobacteriaceae* family and showed that CPCRI-2 was a *Pseudomonadaceae* family member (Fig. S4).  A pie chart distribution generated at each node of the protein taxonomy tree revealed the presence of 4261 shared coding sequences (CDS) between CPCRI-2 and *Pseudomonas* genus. Specifically, 688 CDS are shared between the CPCRI-2 and the genome of the *Pseudomonas putida* species. CPCRI-1 and CPCRI-3 both shared ~4000 CDS with the *Enterobacteriaceae* family.  Additionally, CPCRI-1 shared 1412 CDS with the *Enterobacter* genus and 288 CDS with the *Enterobacter cloacae* species.  However, CPCRI-3 shared only 567 CDS with the *Enterobacter* genus.
